# On the history of abortion from antiquity to the present day, with a focus on Central Europe and Germany

**DOI:** 10.1007/s00404-025-08300-3

**Published:** 2026-01-06

**Authors:** F. M. Dienerowitz, M. David

**Affiliations:** 1 Institute for the History, Theory, and Ethics of Medicine, Mannheim Medical Faculty of Heidelberg University, Mannheim, Germany; 2 Department of Gynecology, Charité – Universitätsmedizin Berlin, Campus Virchow-Klinikum, Augustenburger Platz 1, 13353 Berlin, Germany

**Keywords:** History of abortion, Ethical questions of abortion, Legal background of abortion

## Abstract

The question of how to deal with a pregnancy, whether desired or unwanted, is a complex biological, ethical, social, and medical issue going back for millennia. Every form of regulatory approach to this issue is culturally and temporally specific and is therefore subject to continuous change. Our look at its history and the medical, legal, and religious background begins in ancient times, progresses through history, and ends with a focus on the second half of the nineteenth century and especially the twentieth century in Germany. These ethical, moral, and medical questions are likely to have been discussed in a similar way in other parts of the Western world.

## Introduction

On October 1, 1995, the Pregnancy and Family Assistance Amendment Act came into force in Germany, which, for the past 30 years, has provided the legal framework for the more than 100,000 abortions recorded annually in Germany. According to this act, women can have an abortion without penalty within the first 12 weeks of pregnancy (14 weeks after the last menstrual period) after counseling and a 3-day reflection period (Section 218a, 1 of the German Criminal Code), even though abortion is still classified as illegal and is anchored in the German Criminal Code (StGB **St**raf**g**esetz**b**uch). In addition, it is possible to terminate a pregnancy on medical grounds (Section 218a, 2 of the German Criminal Code) or after sexual abuse (Section 218a, 3). These regulations are seen as a social compromise between protecting the life of the unborn child, its right to life and the pregnant woman’s right to self-determination. In practice, they have proved to be a well-functioning system since the mid-1990s. However, the discussion about abortion and its regulations has been and continues to be the subject of controversy in Germany. In essence, the debate revolves around the question: “How do I as an individual and how does society deal with wanted and unwanted pregnancies?” Answering this twofold question is a complex biological, ethical, social, and medical issue going back thousands of years. Every form of regulatory approach to this issue is culturally and temporally specific and has therefore been subject to continuous discussion and change.

Thus, it is worthwhile to look at the history and the medical, legal, and religious aspects of abortion. Starting with a broad overview of different approaches to the topic in ancient times, we will progress through history, lastly focusing on developments starting in the second half of the nineteenth century and especially the twentieth century, primarily in Germany. Finally, we will touch on the current legal situation in neighboring European countries.

## Antiquity and the Middle ages

Abortion is by no means a modern invention. There are sources dating back to antiquity that refer to abortion. The Indian law book Manava Dharmasastra, which was presumably written between 200 BC and 200 AD, condemns abortion. According to this text, anyone involved in such a crime became unclean, and the woman who aborted her unborn child received the same punishment as someone who killed a Brahmin. Buddhism also prohibited abortion. Any destruction of life was considered a sin, and abortion was punished particularly severely because the souls of unborn children were considered to be malicious and dangerous bloodsuckers. On top of that, it was believed that abortion posed the risk of killing a deceased relative or friend, whose soul was continually being reborn. The Code of Hammurabi, written by the 6th king of Babylon, dates to ancient Babylon and was written around 1750 BC. Among its laws, intended to protect peace and morality in the family, is the threat of a fine for mistreating a (free) woman or killing her unborn child. If the woman died, the perpetrator’s daughter was executed [1].

Few medical records exist from Ancient Egypt. Obstetrics is covered rather sparsely, but the Ebers Papyrus, “the hermetic book on the medicines of the ancient Egyptians” dating back to the sixteenth century BC, provides some information. There are several references to substances which, according to current interpretation, were used as abortifacients for internal and external use. It is not known whether abortion was legally prosecuted, but given the arbitrariness and ruthlessness with which children were killed and abandoned, it is unlikely [1].

Judaism and Greco-Roman culture were particularly significant in the history of abortion in the Western world. In Jewish culture, great value and importance are attached to individual life, in contrast to other cultures and ideologies from the past and present, in which a person’s life is supposed to serve the collective or a higher purpose and can, therefore, be sacrificed. Even though liberal abortion regulations apply in the modern state of Israel, the Torah does not advocate abortion at any point. According to Exodus 21,22, penalties for a miscarriage, resulting from injuring a pregnant woman partly depended on the demands of the aggrieved husband and whether the woman had suffered damage to her health. Further proof of the importance of individual life in Judaism can be found in Psalm 139, 14–16 written by King David: “I praise you, for I am fearfully and wonderfully made. Wonderful are your works; my soul knows it very well. My frame was not hidden from you when I was being made in secret, intricately woven in the depths of the earth. Your eyes saw my unformed substance; in your book were written, every one of them, the days that were formed for me, when as yet there was none of them.” (https://www.die-bibel.de/bibel/NGUE/PSA.139, accessed on August 29, 2025).

Greek views, on the other hand, were shaped by the stance of their philosophers, who had a completely different understanding of biology. Stoicism, for example, considered the conceived child as neither animated (having a soul) nor being human; the embryo was part of the mother’s entrails. If the unborn child was not animated, then its destruction could not be considered an attack on human life and hence, a crime. Abortion was seen as a welcome solution for women who had become pregnant involuntarily, and there was no concern for the rights of the child. Plato and Aristotle, whose ideas were strongly focused on state regulation, advocated abortion for national economic and social reasons, for example, as a means of maintaining ideal population numbers. Plato even called for mandatory abortion for women over 40 years of age. Aristotle, however, emphasized that the fetus must be aborted before it had life and actions, such as movement and sensation—a restriction that would prove significant for the further course of history [1–3].

According to Roman family law, the father of the family had absolute power over all family members and was therefore also authorized to kill the child in the womb. Roman philosophers followed the Stoic school of thought and regarded the fetus as part of the mother—abortion was, therefore, considered a trivial act, to be treated like any other medical procedure. When penalties were imposed, it was not the termination of pregnancy as such that was punished, but the offense that had caused the termination. Historians assume that the rise of Rome led to an increase in abortions. Interestingly, the decline in population numbers in later years is seen as one of the causes of the empire’s downfall. Thus, it can be assumed that legal measures against abortion—such as those introduced under Emperor Septimius Severus (139–211 AD)—were primarily motivated by demographic reasons [2, 4]. The father’s hope for offspring was postulated as the legal interest to be protected, not the right to life of the fetus—even after birth, children were not automatically granted such a right [5].^1^

With the increasing influence of Christianity and its Jewish heritage, abortion lost its social acceptance. This also had to do with the value Christianity placed on human life (rejection of gladiatorial combat, acceptance of abandoned disabled people) and the special protection of the weak. In Christian-Byzantine times, unborn life was respected in the same way as that of adults. At various councils in the 4th to seventh centuries, abortion was subjected to severe ecclesiastical penalties [1].

Nevertheless, Greco-Roman ideas became part of the canonical jurisprudence of the Roman Catholic Church. In accordance with Aristotle’s theory of successive animation, a distinction was made based on the age and sex of the unborn child. Abortions after the animation of the male embryo on the 40th day or the female fetus on the 80th day were considered murder and were punished with excommunication and the death penalty. Earlier abortions were only considered a grave sin in canon law, but were punished less severely [7, 8].

This concept was not abandoned until 1869, following a decree by Pope Pius IX. Since then, the Catholic Church has regarded human life as beginning from the moment of conception and as something to be respected and protected in every way [9]. This shift, based on scientific findings from the second half of the nineteenth century, is also reflected in secular legislation. While the first general German criminal code, the *Constitutio Criminalis Carolina* under Emperor Charles V (1500–1558) in 1532 still distinguished between “living” and “non-living” children in cases of abortion, the *General Land Law for Prussian States* of 1794 granted unborn children “the general rights of humanity” from the moment of conception [10]. In addition to Christian influence and scientific knowledge, demographic reasons also played a role in the various modern criminal prohibitions on abortion. The influential Bavarian Criminal Code of 1813, for example, considered abortion a criminal offense mainly for demographic reasons and not because of any right to life of the embryo or fetus [11–13].

## German Empire (1871–1918)

In 1851, a new criminal code came into force in Prussia, whose provisions on abortion were incorporated, with only slight modifications, into the case law of the North German Confederation (1867) and shortly thereafter, into the Criminal Code of the newly formed German Empire (1871). The classification under Section 218 et seq., in which the illegality of abortion is depicted to this day as a “crime against life”, dates to this period.^2^ The law thus classified abortion as homicide and provided for prison sentences for both the pregnant woman undergoing the abortion and any third parties involved in or performing the act [14]. There were no exceptions to this rule or gradations based on the stage of pregnancy, as these were not up for debate according to the scientific understanding that the embryo was alive from the moment of fertilization [2]. The law remained unchanged until 1926; attempts at reform failed in the turmoil of World War I and the November Revolution of 1918. Until around 1900, discussions about liberalizing the law focused solely on abortion in cases of strict medical indication, i.e., when the life of the pregnant woman was in imminent danger. In these cases, abortions were commonly performed by doctors and accepted from a criminal law perspective. Despite strict legislation and abortion being considered a “moral decline” by politicians, churches, and the medical profession, abortion was not uncommon and rarely led to prosecution. The main reason was most likely that the lower social classes were in dire economic straits and sought a way out either through dangerous and almost unverifiable abortions performed by themselves, or they went to quacks or backstreet abortionists [2].

Opinion started to become much more varied at the beginning of the 1910s, when diverging political movements and opposing ideological beliefs entered the abortion debate. At that time, there were initial, albeit still rare, calls for the complete repeal of Section 218 of the German Criminal Code—mainly from the left-wing political spectrum and from parts of the burgeoning first women’s movement [15]. However, the ideas of racial hygiene driven by social Darwinism gained greater influence [16], spreading among parts of the medical profession as well as in various political camps and finding expression in demands and, in some cases, in the illegal application of eugenic indications for abortion [17]. Ultimately, however, the dominant attitude across the various groups was so-called Pronatalism, which remained the prevailing political opinion in view of the decline in birth rates and, later, the high number of soldiers killed in World War I. Thus, the ban on abortion served as a demographic measure in the interests of the nation state [18].

## Weimar Republic (1919–1933)

The Weimar Republic adopted the provisions of Section 218 et seq. in unchanged form, even though postwar poverty and housing shortages were arguments that led government circles to consider liberalizing contraception and abortion legislation, replacing the pronatalist demand for “offspring for the fatherland.” The high loss of men catalyzed increasing independence for women and promoted the right to self-determination in matters of sexuality and abortion. The October Revolution, i.e., the communist coup in Russia, and its almost unconditional legalization of abortion, encouraged the left-wing political spectrum to demand the same in Germany. This led to various, sometimes far-reaching demands for a reform of Section 218 of the German Criminal Code in the Weimar Republic, both at a political and a societal level. As early as 1920, a motion by the SPD (Social Democratic Party) called for the decriminalization of abortions within the first 3 months of pregnancy. The KPD (Communist Party of Germany) went even further in its demands and fought for the complete repeal of Section 218 with slogans such as “Your body belongs to you.” Towards the end of the 1920s, these demands led to a broad social movement that was widely covered in the press, including protests, self-accusation campaigns, and cultural events, mainly supported by the left-wing movements [2, 18].^3^

In terms of Realpolitik, however, the views of those in favor of changing the regulations on abortion were too far apart, so that despite numerous motions, no fundamental reform took place. Another reason was that, despite all the effective publicity activities, large sections of society, as well as the conservative parties continued to reject abortion. As a result, in a reformulation of the law in 1926 following an initiative by the SPD (Social Democratic Party), only the penalties were significantly reduced. A medical indication, which was narrowly defined, was accepted by society at large and was legitimized in a 1927 ruling by the Reichsgericht (Supreme Court of the Reich) [20]. This gave the still predominantly conservative medical profession a certain degree of legal security when performing abortions in cases where the mother’s health was at risk [2, 18, 21].

## National Socialism—the Nazi era (1933–1945)

With the decline of the Weimar Republic, further reform efforts failed and the social movement ebbed away. When the National Socialists came to power, the counseling centers that had emerged in the 1920s and were run by numerous associations and organizations for sexual reform and who, among other things, referred women seeking abortions to doctors, were closed or converted into counseling centers that were ideologically loyal to the regime. In addition, leading figures of the sexual reform movement were arrested. An important element of Nazi ideology was the concept of racial hygiene and eugenics, which had already begun to emerge during the German Empire and gained widespread popularity during the Weimar Republic, paving the way for its consistent implementation under Hitler well before 1933 [2, 17]. Under the National Socialists, part of the liberalization of abortion legislation passed in 1926 was repealed in 1933 [22], and the possibilities for illegal abortions of healthy children were increasingly reduced through strict control mechanisms within the state health care system [23].

In 1935, medical and eugenic indications for abortion were introduced into the German Criminal Code for the first time by extending the *Law for the Prevention of Hereditary Diseases*, a law on compulsory sterilization motivated by eugenic and social Darwinist ideas that had been enacted 2 years earlier [24]. Since its implementation was placed in the hands of the medical profession, thus increasing its demographic influence, many doctors welcomed this development or even promoted it. In addition, “pregnant women of foreign descent” were encouraged to have abortions on racial grounds. From 1940 onwards, a secret decree allowed women to be forced to terminate their pregnancies if the unborn child was suspected of having a hereditary disease or for reasons of racial hygiene [23]. Overall, it is clear that the National Socialists were not concerned with protecting the individual when it came to pregnancy, but rather with implementing an ideology. The eradication of “undesirable” life in the womb was pursued, but abortions that harmed the “German national strength,” were severely punished.^4^

From 1943 onwards, the *Ordinance for the Protection of Marriage, Family, and Motherhood* threatened the death penalty for abortions of healthy German children; abortions of “non-Aryan” women, on the other hand, were exempt from punishment [25]. Towards the end of the Second World War, another decree was issued to prevent “racially inferior” offspring in pregnancies resulting from rape by Soviet soldiers [2, 21].

## Occupation period (1945–1949)

After the war, the military governments of the victorious powers repealed all of the stricter penalties enacted after 1933, including the amendments to Section 218. However, the details of the legal situation regarding abortion differed in the four occupation zones, with the trend toward regulations regarding indications for an abortion, already discussed during the Weimar Republic, gaining ground. In the three western zones, abortion was permitted on various grounds, including medical reasons and—in view of the mass rapes committed by soldiers of the victorious powers—criminal reasons. In the Soviet occupation zone, the regulations were initially even more liberal; some state laws included eugenic and social indications in addition to medical ones [2, 21].

The majority of doctors in West Germany supported abortions for medical reasons only, but departing from the strict medical indications that had been propagated up to that point, social hardship and rape were now also recognized as influential factors and were routinely taken into account in practice [19]. At the political level, the KPD (Communist Party of Germany) and parts of the SPD (Social Democratic Party) campaigned in the media and at rallies for the legalization of abortion, as they had done during the Weimar Republic, while the CDU (Christian Democratic Union) advocated the fundamental protection of unborn life. However, in view of the more existential challenges of the post-war period, society showed little interest in a possible amendment to Section 218 of the German Criminal Code, which is why the issue faded into the background in public debate until the end of the 1960s [2].

## Federal Republic of Germany (FRG) and German Democratic Republic (GDR) (1949–1990)

On May 23, 1949, the Basic (Constitutional) Law for the Federal Republic of Germany (i.e. West Germany) was promulgated. This law and its interpretation—in particular Article 1, paragraph 1^5^ and Article 2, paragraph 2^6^—were to become fundamental to how abortion was handled legally in the further course of the Federal Republic’s history. Initially, there was little social pressure on politicians to change the law, because in the 2 decades after the end of the Second World War, there was a return to conservative sexual morality associated with a negative attitude towards abortion [2]. However, this changed in the late 1960s with the so-called sexual revolution, the student movement, and the emergence of the second feminist movement. These groups not only had a positive attitude toward birth control and sexual freedom, but also proclaimed abortion as a woman’s right and freedom, and regarded it as an extended form of contraception.^7^ Various protests were held to campaign for the complete repeal of Section 218; any rights of the unborn child were not considered [27, 28].

Following the social-liberal coalition formed by the SPD (Social Democratic Party) and FDP (Free Democratic Party) in the fall of 1969, these demands were finally picked up by politicians. After extensive socio-political debate, the majority of the German Bundestag (Lower House of the German Federal Parliament) voted in 1974 in favor of a new regulation granting general immunity from prosecution in the first 3 months of pregnancy after having received prior consultation. However, because the CDU/CSU (Christian Democratic Union/Christian Social Union) parliamentary group and some of the states governed by the Union parties lodged an appeal with the Federal Constitutional Court, the law could not come into force. In the so-called first deadline ruling, the court ruled in favor of the Union parties on February 25, 1975. The deadline regulation was considered incompatible with the German Basic (Constitutional) Law, as unborn life also had the right to life, and this right was not guaranteed in the case of a deadline solution within the first 3 months. The court also considered the planned counseling of pregnant women to be unsuitable for encouraging them to continue with their pregnancies. However, the ruling recognized immunity from prosecution in exceptional cases, such as medical, criminal, and child-related (eugenic) indications; even a general (social) hardship indication was declared acceptable. Ultimately, a draft bill drawn up by the governing parties SPD and FDP was passed in 1976—a far-reaching indication solution with exemption from punishment in cases of medical, criminal, eugenic, and social (hardship) indications, whereby immunity from prosecution was limited to 22 weeks after conception in the case of eugenic indications and to 12 weeks after conception in the case of criminal and social hardship indications [21, 28].

Reactions to this legal compromise varied; the left-wing women’s movement was disappointed because its goal of complete self-determination for women over their bodies had not been achieved. The Catholic Church also criticized the new legislation. To this day, abortion is not accepted or practiced in Catholic hospitals, except for very narrow vital medical indications, where the life of the mother is at risk. The medical profession increasingly accepted the reform, even though in 1973 most doctors had rejected a deadline regulation.^8^ The broad interpretation of medical indications for abortion that was already widely practiced now had a firm legal basis. Departing from their previous restrictive stance, doctors increasingly performed abortions based on social hardship. However, this was at odds with the reality of German prosperity and the well-developed welfare state, and was sometimes misused as a “back door” to a deadline regulation [2, 30].

In the GDR (German Democratic Republic, i.e. East Germany), which was founded in 1949, abortion legislation also underwent several changes, but these were motivated by a different set of values and norms. In 1950, due to a massive increase in legal abortions, the very liberal regulations of the Soviet occupation zone were narrowed down to medical and eugenic reasons in the interests of a qualitative and quantitative population policy. In the 1960s, the grounds for abortion were expanded to include socio-medical indications. In 1972, there was another reform. A deadline regulation was established, which gave women the right to terminate their pregnancy within the first 3 months of pregnancy, and termination remained non-punishable for the woman even in later weeks of pregnancy. This regulation was intended to promote the ideological goal of enforcing equal rights and did not consider the protection of the unborn child’s life [2, 31].

## Reunified Germany (1990 to present)

There was reason to revise Section 218 of the German Criminal Code again after 1990 due to German reunification, with two different regulations regarding abortion in the former GDR (East Germany) and the Federal Republic of Germany (West Germany) clashing with each other. Over the years, the different regulations had not only shaped society’s attitude toward abortion, but also contributed to different abortion rates: In West Germany about 11% of all pregnancies were terminated, in East Germany the figure was almost 30%. Thus, a new law for the whole of Germany had to be drawn up [5].

In 1992, after difficult negotiations, a compromise across party lines was passed: A deadline regulation, which, among other things, stipulated that abortion in the first 12 weeks after fertilization would be legal. This again led to a lawsuit before the Federal Constitutional Court, which declared parts of the new regulation unconstitutional in its ruling of May 28, 1993. In its second ruling on the deadline regulation, the court again declared that according to the Basic (Constitutional) Law, abortion was to be considered illegal at any time on the basis of the right to life and human dignity of the unborn child. However, in contrast to the 1975 ruling, a deadline regulation with immunity from prosecution for abortion in the first 12 weeks after conception was now approved, provided the pregnant woman had received prior counseling. It was hoped that the woman’s autonomous decision and the help provided during counseling would improve protection for the unborn child [32, 33]. After various draft bills, the Pregnancy and Family Assistance Amendment Act mentioned in the introduction was passed by a clear majority on June 29, 1995. In practice, a close network of counseling centers run by various organizations has been established, and abortion facilities can be found almost everywhere in Germany. Since then, the abortion rate has been between 11 and 16%, which corresponds to 95,000–135,000 recorded abortions per year [34]. If statistics are correct, that number is—compared to other European countries with even more liberal regulations—a rather low abortion rate. Thus, the concept of mandatory counseling helps women to decide to keep the child, even though little is known about the reasons for abortion. The few studies on that subject indicate that often partnership and external pressure, overburdening, material needs, and unfavorable timing of the pregnancy are the main reasons for pregnancy conflict [5, 34, 35].

## European perspectives and concluding remarks

The latest developments in Germany should not be viewed in isolation. Moral and medical issues, such as access to abortion facilities, are also being discussed in a similarly controversial manner in other countries in the Western world [36]. In many formerly predominantly Christian countries, the criminal prohibition of killing unborn life is being transformed into a “right to abortion” following the somewhat vague principle of “reproductive self-determination”. This is in line with the positions of the United Nations and the European Parliament; the latter called for the inclusion of such a right in the Charter of Fundamental Rights of the European Union in July 2022 and April 2024. In France, for example, the “freedom to have an abortion” was enshrined in the constitution in March 2024, in Denmark, the deadline for abortions was extended from 12 to 18 weeks of pregnancy and in 2025, in England, it was even extended from 24 weeks of pregnancy to birth. These developments are, among other things, a consequence of the social upheavals of the 1960s. The demands of the youth back then are now being incorporated into today’s political decisions. This is contrasted by an increasingly resurgent right-wing conservative movement in many Western countries, which tends to view abortion critically [34].

In a way, politics and society in the West could be seen as engaged in a kind of culture war, which is particularly evident in the political issue of “abortion”—and perhaps can only be understood from a historical perspective. While one side, following the Stoic Greek view, regards the unborn child as part of the mother without rights of its own (“my womb belongs to me”), the other side fights for the Judeo-Christian understanding that attributes independent value to life even before birth. There seems to be no real resolution of these two positions, only a fragile compromise, as German legislation attempted to achieve 30 years ago.

## Data Availability

Not applicable.

## Footnotes

1 In addition to abortion, infanticide was also a recognized means of birth control in ancient cultures—except in Judaism—until Emperor Constantine the Great banned the killing of children throughout the empire in 318 in accordance with Judeo-Christian beliefs [6].

2 The prohibition of abortion can be found in Section 16 (Crimes against life) of the Special Part of the German Criminal Code, alongside the sections on murder (Section 211) and manslaughter (Section 212).

3 The play “Cyankali” by the physician Friedrich Wolf, which was later made into a film, became famous for its poignant portrayal of the fates of pregnant working-class women. It met with such a strong response that it ultimately triggered the “largest public mobilization against § 218 in the Weimar Republic” [19].

4 This is also evident in the fact that the ban on abortion in the Criminal Code was to be removed from the section on homicide and incorporated into the new section “Attacks on race and genetic material.” Although this had not happened by 1945, it illustrates that the ban on abortion was not understood as individual protection, but as an instrument of state population and race policy [2].

5 “Human dignity shall be inviolable. To respect and protect it shall be the duty of all state authority.”

6 “Every person shall have the right to life and physical integrity. Freedom of the person shall be inviolable. These rights may be interfered with only pursuant to a law.”

7 It can be observed that there is a close link between the discussions on contraception and abortion. The liberalization of contraception often led to a relaxation of abortion laws and becoming a topic of political and social debate [26].

8 A resolution of the German Medical Association stated 1973 that there is no period in the development of human life after implantation in the womb before which the unborn human life deserves no or less protection [29].
